# School refusal and bullying in children with autism spectrum disorder

**DOI:** 10.1186/s13034-020-00325-7

**Published:** 2020-05-07

**Authors:** Marina Ochi, Kentaro Kawabe, Shinichiro Ochi, Tomoe Miyama, Fumie Horiuchi, Shu-ichi Ueno

**Affiliations:** 1 Department of Neuropsychiatry, Ehime University Graduate School of Medicine, Toon, Ehime 791-0295 Japan; 2 Horie Hospital, Matsuyama, Ehime 791-0295 Japan; 3 Center for Child Health, Behavior and Development, Ehime University Hospital, Toon, Japan; 4 Matsuyama Kinen Hospital, Matsuyama, Ehime 791-0295 Japan

**Keywords:** Autism spectrum disorder, Bullying, Neurodevelopmental disorders, Outpatients, School refusal

## Abstract

**Background:**

Few studies have explored school refusal in children with autism spectrum disorder (ASD), despite being considered a serious problem. One of the leading causes of school refusal is bullying, which is defined by the feelings of students who are bullied or not, and psychological suffering caused by a psychological or physical attack. This study investigated the characteristics of school refusal in children with ASD.

**Methods:**

A total of 94 outpatients with school refusal and ASD and 143 outpatients with school refusal without ASD aged 6–18 years were included. Chi squared tests and Mann–Whitney tests were used to compare the characteristics of school refusal in children with and without ASD. Univariate and multivariate logistic regression analyses were performed to analyze the reasons for school refusal in children with ASD by sex.

**Results:**

School refusal significantly occurred earlier in children with ASD than in those without. In addition, “bullying” was significantly associated with school refusal in both boys and girls with ASD.

**Conclusions:**

These findings suggest that school refusal should be monitored early in children with ASD. The importance of recognizing bullying among children with ASD should be highlighted as an opportunity for early intervention.

## Background

School refusal is an increasingly serious issue among children. The term “school refusal” coalesces outdated terms such as truancy, school avoidance, school absenteeism, and school phobia. In 1941, the term “school phobia” first was introduced in clinical literature by Johnson [1]. School refusal refers to a child’s refusal to attend school, as well as difficulties with remaining in school for an entire day, including missing entire or partial school days, skipping classes, or unjustifiably arriving late [2]. Moreover, school refusal refers to a child’s inability to continue school for mental health reasons, such as anxiety and depression [3]. According to the National Association of School Psychologists, individuals who avoid school are more likely to have long-term emotional issues, such as depression and anxiety, poor academic achievement, dropping out of school, and suicide [3, 4]. School refusal is also a key risk factor for violence, injury, substance use, psychiatric disorders, and economic deprivation [5]. The prevalence of school refusal has been reported to be approximately 1% in school-aged children, which is similar among both sexes [6]. In Japan, the educational system is under the authority of the Ministry of Education, Culture, Sports, Science, and Technology (MEXT), which defines school refusal as the students’ lack of attendance for > 30 days per year for reasons other than sickness or economic causes, including psychological, emotional, physical, or social reasons [7]. Currently, the number of students with school refusal is an increasingly serious issue in Japanese education. The total number of students in elementary and junior high schools has decreased from approximately 13 million in 1995 to 10 million in 2016 due to low birth rate. Conversely, the number of students with school refusal has increased from 81,591 in 1995 to 133,683 in 2016. Therefore, the rate of school refusal was > 1% in Japanese elementary and junior high school students in 2016 [8]. Japanese children and adolescents aged 6–15 years are required to compulsorily attend school.

Reasons for school refusal are complicated and include biological, psychological, and social factors. Biological factors that impact school refusal include neurodevelopmental disorders, such as autism spectrum disorder (ASD) [9]. ASD is a lifelong set of heterogeneous neurodevelopmental disorders characterized by developmental delays in social communication and repetitive behaviors [10]. Some studies revealed that the rate of school refusal in children with ASD was significantly more than in children with typical development [9, 11]. A markedly skewed sex distribution has been consistently reported in ASD, despite the recently improved recognition of autism in girls [12]; the ratio is still estimated to be around 2–3 (boys):1 (girls) [12, 13]. Wang et al. reported that girls with ASD show greater socio-emotional reciprocity, and nonverbal girls suffer increased communication impairment compared with boys [14]. Differences might exist in the underlying etiology and symptom presentation of ASD among girls, which could be associated with both reduced risk of developing ASD, as well as failure of recognizing ASD in girls [15]. School refusal associated with ASD might have different characteristics according to sex, although there are no reports of sex difference in this population.

One of the leading causes of school refusal is bullying [16]. Olweus defined bullying as an aggressive behavior that is repetitive, intentional, and physically or emotionally hurtful [17]. In Japan, MEXT uses two points to define bullying: (1) “We must judge not ceremonially but from the feelings of students who are bullied whether there is bullying or not” and (2) “Bullying is defined as those who are in relationships with someone and feel psychologically damaged because of a psychological or physical attack” [18]. Research indicates that children with ASD are at a greater risk for being bullied victims and/or perpetrators [19–21]. Previous studies in the USA, Canada, the UK, and the Netherlands reported bullying prevalence ranging from 7 to 75% for individuals with ASD as victims and from 19 to 46% as perpetrators [19, 22–24]. Children with ASD are unable to develop the theory of mind (ToM), which is the ability to comprehend and describe mental states such as belief, intention, and emotion in themselves and others [25], as they have difficulty understanding the concept of false belief.

Bullying might be one of the factors that lead to school refusal for students with ASD who have less developed ToM, which often leads to isolation and loneliness [22], and adolescents with ASD are more likely socially withdrawn than TD individuals [26], which might result in school refusal. In clinical settings, the school refusal rate was 12.2–28.6% in students attending Japanese schools [27–29]. Although studies indicate that school refusal is a considerable problem in students with ASD, detailed studies observing an association between school refusal and ASD-related factors have not been performed. Moreover, the school education curriculum considerably varied between countries, and overseas data or papers were not as informative.

This study compared school refusal between children with and without ASD. Accordingly, we hypothesized that the characteristics of school refusal in children with ASD are different from those in children without ASD.

## Methods

### Participants and study design

This study was approved by the ethics committee of Ehime University Graduate School of Medicine (IRB No. 1507007). Written informed consent was obtained from the children’s caregivers. This was a retrospective chart review study that covered the records of all psychiatric outpatients at Ehime University Hospital, Japan, between January 2012 and December 2016. Participants were required to meet the inclusion criteria for this study: (1) age, 6–18 years; (2) having school refusal according to the MEXT definition: more than 30 days per year; (3) currently studying in an inclusive classroom but not being pulled out to special education room. Inclusive classroom and special education room have different systems and situations; therefore, students in special classes were excluded from this study. Participants were divided into two groups: school refusal students with ASD and without ASD. ASD was diagnosed by board-certified child psychiatrists based on the Diagnostic and Statistical Manual of Mental Disorders-5 (DSM-5) criteria: deficits in social-emotional reciprocity, nonverbal communicative behaviors, stereotypical or repetitive motor movements, inflexible adherence to routines, and hyper- or hyporeactivity to sensory input or unusual interests in sensory environmental aspects [30]. Characteristics that lead to school refusal, including age at onset of school refusal, having siblings, having parents or a single parent, and taking psychotropic drugs were elucidated through interviews from students and their parents and school teachers, if possible. The psychosocial function of children during the first hospital visit was evaluated using the Children’s Global Assessment Scale (CGAS) [31], ranging from 0 to 100, with higher scores indicating better function, evaluated using the most impaired functioning level in the last 1 month. Reasons for school refusal were categorized using MEXT-specified items: (1) bullying, (2) friendship other than bullying, (3) problems with school personnel, (4) problems with school record, (5) concern about the course, (6) problems with school regulations, (7) maladjustment at the time of school entrance or promotion, (8) rapid changes in the living environment, (9) parent–child relationship problems, (10) home discord, (11) absence due to illness, (12) delinquency, (13) lethargy, (14) anxiety and emotional confusion, (15) intentional denial, (16) other reasons (physical symptoms), and (17) unknown. These data were assessed by outpatient clinicians.

### Sample size

The sample size for the means of the two groups was calculated using G* power 3.1.9.2 software. A size effect of 0.5, significance level of α = 0.05, and statistical power of 1 − β = 0.95 were considered, and a power analysis indicated the need for 184 participants. The necessary sample size was fulfilled in this study.

### Data analysis

Chi squared tests were used to analyze categorical variables. Mann–Whitney tests were used to compare characteristics of participants with and without ASD. The variables used were sex, age at onset of school refusal, CGAS, duration between the onset of school refusal and first hospital visit, presence of siblings, presence of a single parent or parents, and psychotropic drug therapy status. Multiple regression analysis was used to identify primary factors affecting school refusal. Univariate and multivariate logistic regression analyses were performed to analyze the reasons of school refusal in children with ASD as well as its association with sex. All values are presented as mean ± standard deviation. P < 0.05 was considered as statistically significant.

## Results

### Comparison of characteristics of participants with and without ASD

Demographic data for each group of participants are shown in Table 1. Among 237 participants with school refusal, 94 were diagnosed with ASD, whereas 143 had no ASD. The percentage of boys was significantly higher in participants with ASD than in those without (P < 0.001). The mean age at onset of school refusal was 12.6 ± 2.2 years in students with ASD, which was significantly younger than those without ASD (13.8 ± 2.1 years; P < 0.001). CGAS scores were significantly lower in participants with ASD than in those without (P < 0.05). None of children was diagnosed with anxiety or depressive disorders. Two children without ASD were diagnosed with obsessive compulsive disorder (OCD), two were diagnosed with eating disorders (ED), and one was diagnosed with brief psychiatric disorder. One child with ASD was co-morbid with OCD and ED. No significant differences were observed between participants with ASD and without ASD with respect to the duration between the onset of school refusal and the first hospital visit, presence of siblings, presence of single parent or parents, or psychotropic drug therapy status.

**Table 1:** Comparison of characteristics of participants with and without ASD

	ASD (+) n = 94 n (%)	ASD (−) n = 143	P value
Gender			
Boy	67 (71.3)	49 (34.3)	< 0.001**
Girl	27 (28.7)	94 (65.7)	
Age at onset of school refusal (y), mean ± SD	12.6 ± 2.2	13.8 ± 2.1	< 0.001 ^b^
CGAS, mean ± SD	43.6 ± 7.0	45.5 ± 7.5	0.036 ^a^
Duration between onset of school refusal and the first visit to hospital (months), mean ± SD	8.9 ± 14.2	9.6 ± 13.9	0.531
Having sibling			
Yes	70 (74.5)	115 (80.4)	0.428
No	22 (23.4)	28 (19.6)	
Unknown	2 (2.1)		
Parents			
Single	17 (18.1)	31 (21.7)	0.501
Both	77 (81.9)	112 (78.3)	
Taking psychotropic drugs			
Yes	20 (21.3)	32 (22.4)	0.841
No	74 (78.7)	111 (77.6)	

Chi squared test, *p < 0.05, **p < 0.001

Mann–Whitney test, ^a^p < 0.05,^b^p < 0.001

Multiple regression analysis revealed that ASD was significantly associated with sex (B = 0.329, β = 0.337, P < 0.001), age at onset of school refusal (B = 0.005, β = 0.265, P < 0.001), and CGAS score (B = 0.010, β = 0.153, P < 0.05). However, the presence of ASD was not significantly associated with the duration between the onset of school refusal and first hospital visit (B = 0.002, β = 0.071, P > 0.05), presence of a sibling (B = 0.069, β = 0.057, P > 0.05), presence of single parent or parents (B = 0.032, β = 0.026, P > 0.05), or psychotropic drug therapy status (B = 0.026, β = 0.022, P > 0.05).

### Reasons for school refusal among children with ASD according to sex

The results of univariate and multivariate logistic regressions analyzing the reasons for school refusal in boys with and without ASD are shown in Table 2 and those in girls with and without ASD are also shown in Table 3. As shown in Table 2, univariate logistic regression analyses revealed that bullying tended to be significantly associated with school refusal in boys with ASD (P = 0.054). Using a multivariate model of school refusal in boys with ASD as the dependent variable, we found that bullying tended to be significantly associated (P = 0.058). As shown in Table 3, univariate logistic regression analyses revealed that two variables, maladjustment during school entry or promotions, and other reasons, were significantly associated with school refusal in girls with ASD (P = 0.004, P = 0.006). Using a multivariate model of school refusal in girls with ASD as the dependent variable, we found that bullying, maladjustment during school entry or promotions, and other reasons were significant factors (P = 0.005, P = 0.005, P = 0.002).

**Table 2:** Reasons for school refusal in boys with ASD

	Univariate			Multivariate		
	Odds ratio	95% CI	P-value	Odds ratio	95% CI	P-value
1. Bullying	4.616	0.974–21.873	0.054	4.518	0.953–21.420	0.058
2. Friendship other than bullying	1.437	0.658–3.140	0.363	–	–	–
3. Problems with school personnel	2.204	0.839–5.788	0.109	–	–	–
4. Problems with school record	0.604	0.248–1.471	0.267	–	–	–
5. Concern about course	0.719	0.139–3.722	0.694	–	–	–
6. Problems with school regulations	1.237	0.281–5.439	0.779	–	–	–
7. Maladjustment at the time of school entrance or promotion	1.585	0.587–4.280	0.364	–	–	–
8. Rapid changes in living environment	0.381	0.105–1.383	0.142	–	–	–
9. Problems in parent–child relationship	1.312	0.569–3.025	0.524	–	–	–
10. Discord at home	0.413	0.094–1.816	0.242	–	–	–
11. Absence due to illness	1.053	0.370–2.993	0.923	–	–	–
12. Delinquency	1.895	0.352–10.200	0.457	–	–	–
13. Lethargy	0.559	0.142–2.199	0.405	–	–	–
14. Anxiety and emotional confusion	1.66	0.695–3.960	0.254	–	–	–
15. Intentional denial	1.46	0.256–8.315	0.67	–	–	–
16. Others (physical symptoms, etc.)	1.574	0.730–3.393	0.247	–	–	–
17. Unknown	0.346	0.061–1.971	0.232	–	–	–

**Table 3:** Reasons for school refusal in girls with ASD

	Univariate			Multivariate		
	Odds ratio	95% CI	P-value	Odds ratio	95% CI	P-value
1. Bullying	2.443	0.727–8.206	0.148	7.914	1.842–34.003	0.005*
2. Friendship other than bullying	1.454	0.616–3.431	0.393	–	–	–
3. Problems with school personnel	1.461	0.419–5.088	0.552	–	–	–
4. Problems with school record	0.487	0.132–1.790	0.278	–	–	–
5. Concern about course	0.672	0.138–3.271	0.622	–	–	–
6. Problems with school regulations	0	0	0.999	–	–	–
7. Maladjustment at the time of school entrance or promotion	4.722	1.645–13.557	0.004*	5.385	1.670–17.369	0.005*
8. Rapid changes in living environment	2.825	0.818–9.754	0.101	–	–	–
9. Problems in parent–child relationship	1.3	0.502–3.363	0.589	–	–	–
10. Discord at home	0.916	0.277–3.031	0.886	–	–	–
11. Absence due to illness	0.994	0.194–5.091	0.995	–	–	–
12. Delinquency	0.8–65	0.093–8.083	0.899	–	–	–
13. Lethargy	2.427	0.384–15.327	0.346	–	–	–
14. Anxiety and emotional confusion	0.54	0.224–1.302	0.17	–	–	–
15. Intentional denial	0.714	0.189–2.695	0.619	–	–	–
16. Others (physical symptoms, etc.)	3.45	1.422–8.370	0.006*	5.314	1.835–15.391	0.002*
17. Unknown	1.769	0.154–20.291	0.647	–	–	–

*P < 0.05

## Discussion

This study showed that the age of onset of school refusal was 12.6 ± 2.2 years in children with ASD, which is earlier than 13.8 ± 2.1 years in children without ASD. Previous studies have indicated that the age at onset of school refusal for Japanese children with ASD was 10.6 ± 3.9 years; however, their results were not compared with those of children with typical development [11]. School refusal is considered as a heterogeneous, complex condition caused and maintained by multiple interrelated factors. The cause of school refusal associated with ASD might be different. Two factors might affect school refusal among children with ASD. First, participants with ASD are directly influenced by school-related stress factors, such as bullying, poor academic performance, and changes in the class or teachers. Munkhaugen et al. speculated that students with ASD are especially vulnerable to stressful emotional events in coping with school situations [32]. Second, participants may wish to avoid something unpleasant. Atypical responses or avoidance of eye contact in ASD can occur unconsciously, and these responses may be based on mechanisms of impaired social functioning [33]. These facts suggest that school refusal in children with ASD may occur at a younger age than in those without ASD. Therefore, school refusal, particularly during elementary school, should be examined considering their neurodevelopmental characteristics. In summary, this study indicated that the onset of school refusal tended to be early among children with ASD.

A previous study has reported that bullying is one of primary factors for school refusal [11]. Furthermore, participants with ASD experience bullying at school more often than those without [20, 34–36]. Children with ASD display considerable difficulty in reciprocal social interactions with impaired social communication skills [37]. There are interactive interpretations on bullying for children with ASD. Children with ASD may believe that peers are bullying them when this is not happening or children with ASD may interpret that peers are not bulling them when this is happening [37, 38]. These difficulties can contribute to isolation from peers. Further, repetitive and ritualistic behaviors and restricted interests make children with ASD “stand-out” among their peers [39]. These characteristics promote children with ASD to feel ridiculed [19]. In this study, bullying was significantly associated with school refusal in both boys and girls with ASD. Van Roekel et al. have reported that the prevalence of bullying in children with ASD is higher in general classes than that in special education classes and that transferring to special education classes may reduce the prevalence of bullying [37]. This study also focused on children with ASD studying in inclusive classes. A study indicated that bullying victimization can lead to increased anxiety in patients with ASD [40]. Furthermore, children with ASD having symptoms of anxiety and depression showed increased risks of victimization [41]. Bullying is related to the later development of psychopathology [42]. Therefore, clinical psychiatrists should pay careful attention to children with ASD who refuse school because of bullying.

In terms of girls with ASD, maladjustment during school entry or promotions and physical symptoms were significantly associated with school refusal. The transition from primary to secondary school is considered challenging for students with ASD [42]. The time of school entrance or promotions to children with ASD may be a major stress and may trigger the school refusal. Furthermore, students with ASD may experience difficulty in expressing their emotions regarding situations linked to the school setting [43]. Children with ASD often cannot directly express their anxiety and emotional confusion. They might express their feelings as physical symptoms such as headaches and nausea. Girls with ASD are reported to be more likely to experience a lack of diagnosis, delay in diagnosis, or misdiagnosis than boys with ASD [14]. In general, it is difficult for girls with ASD to grasp developmental characteristics and they may experience increased stress; therefore, it is vital to carefully diagnose ASD in girls than in boys.

## Limitations

This study has several limitations. First, 71.3% of participants in the ASD group and 34.3% in the non-ASD group were boys, which might show differences between sexes rather than ASD/non-ASD. Our data could not completely compare characteristics of school refusal between the ASD and non-ASD group. Second, age was a relatively wide range. Therefore, characteristics of school refusal should be characterized according to the following age groups: 6–9, 9–12, 12–15 years, and 15–18 years, respectively. Third, participants did not undergo intelligence quotient (IQ) test. IQ differences might be related to some issues, such as difficulty in learning and poor communication due to delayed verbal development, which may allow the identification of different reasons for school refusal with or without intellectual issues. Fourth, confounding factors, such as domestic environment, including economic status, were not controlled. Therefore, future studies should investigate the effects of IQ, restricted age ranges, and domestic environment on school refusal.

## Conclusion

This study shows that bullying may contribute to school refusal in students with ASD. To prevent school refusal in children with ASD, school teachers must take extreme care to prevent bullying of students with ASD. Early diagnosis and intervention for ASD might prevent school refusal in children with ASD.

## Data Availability

Not applicable.

## Abbreviations

ASD: Autism spectrum disorder
CGAS: Children’s Global Assessment Scale
IQ: Intelligence quotient
